# Group B *Streptococcus* Induces Neutrophil Recruitment to Gestational Tissues and Elaboration of Extracellular Traps and Nutritional Immunity

**DOI:** 10.3389/fcimb.2017.00019

**Published:** 2017-02-03

**Authors:** Vishesh Kothary, Ryan S. Doster, Lisa M. Rogers, Leslie A. Kirk, Kelli L. Boyd, Joann Romano-Keeler, Kathryn P. Haley, Shannon D. Manning, David M. Aronoff, Jennifer A. Gaddy

**Affiliations:** ^1^Department of Medicine, Vanderbilt University School of MedicineNashville, TN, USA; ^2^Department of Medicine, Vanderbilt University Medical CenterNashville, TN, USA; ^3^Department of Pathology, Microbiology and Immunology, Vanderbilt University Medical CenterNashville, TN, USA; ^4^Department of Pediatrics, Vanderbilt University Medical CenterNashville, TN, USA; ^5^Department of Biomedical Sciences, Grand Valley State UniversityGrand Rapids, MI, USA; ^6^Department of Microbiology and Molecular Genetics, Michigan State UniversityEast Lansing, MI, USA; ^7^Department of Veterans Affairs, Tennessee Valley Healthcare SystemsNashville, TN, USA

**Keywords:** *Streptococcus agalactiae*, group B *Streptococcus*, pregnancy, neutrophils, metal

## Abstract

*Streptococcus agalactiae*, or Group B *Streptococcus* (GBS), is a gram-positive bacterial pathogen associated with infection during pregnancy and is a major cause of morbidity and mortality in neonates. Infection of the extraplacental membranes surrounding the developing fetus, a condition known as chorioamnionitis, is characterized histopathologically by profound infiltration of polymorphonuclear cells (PMNs, neutrophils) and greatly increases the risk for preterm labor, stillbirth, or neonatal GBS infection. The advent of animal models of chorioamnionitis provides a powerful tool to study host-pathogen relationships *in vivo* and *ex vivo*. The purpose of this study was to evaluate the innate immune response elicited by GBS and evaluate how antimicrobial strategies elaborated by these innate immune cells affect bacteria. Our work using a mouse model of GBS ascending vaginal infection during pregnancy reveals that clinically isolated GBS has the capacity to invade reproductive tissues and elicit host immune responses including infiltration of PMNs within the choriodecidua and placenta during infection, mirroring the human condition. Upon interacting with GBS, murine neutrophils elaborate DNA-containing extracellular traps, which immobilize GBS and are studded with antimicrobial molecules including lactoferrin. Exposure of GBS to holo- or apo-forms of lactoferrin reveals that the iron-sequestration activity of lactoferrin represses GBS growth and viability in a dose-dependent manner. Together, these data indicate that the mouse model of ascending infection is a useful tool to recapitulate human models of GBS infection during pregnancy. Furthermore, this work reveals that neutrophil extracellular traps ensnare GBS and repress bacterial growth via deposition of antimicrobial molecules, which drive nutritional immunity via metal sequestration strategies.

## Introduction

### GBS and pregnancy

*Streptococcus agalactiae* (Group B *Streptococcus*, GBS) is a leading cause of adverse pregnancy and neonatal outcomes including stillbirth, chorioamnionitis, preterm birth, and neonatal sepsis and meningitis (Verani et al., 2010; Koumans et al., 2012; Kwatra et al., 2014; Kim et al., 2015). While up to 50% of pregnant women screen positive for GBS rectovaginal colonization at some point during pregnancy, most women maintain asymptomatic colonization (Kwatra et al., 2014). However, in some neonates, invasive GBS disease occurs via vertical transmission either from ascending vaginal infection during pregnancy or from exposure during vaginal delivery, resulting in lifelong health impairments for affected children (McNamara et al., 1997; Verani et al., 2010; Koumans et al., 2012; Kwatra et al., 2014; Kim et al., 2015). Consequently, the CDC recommends routine GBS screening during late pregnancy and antibiotic prophylaxis for those testing positive (Koumans et al., 2012). Despite this intervention, GBS remains the leading infectious cause of morbidity and mortality among neonates in the United States (Verani et al., 2010).

### Chorioamnionitis and neutrophils

Ascending bacterial infection from the lower genital tract to the uterine cavity is the most common cause of intra-amniotic infection, which leads to profound inflammation of the extraplacental membranes surrounding the fetus; a process that is termed chorioamnionitis (McNamara et al., 1997; Kim et al., 2015). Ascending microorganisms are first localized in the supracervical decidua; subsequent propagation and chorioamniotic passage leads to microbial infection of the amniotic cavity and even the fetus (Kim et al., 2015). Microbial invasion of the amniotic cavity has been shown to induce a robust inflammatory response including an increase in pro-inflammatory cytokines such as IL-1, TNF-alpha, IL-6, and IL-8, as well as a dramatic increase in neutrophil count (Kim et al., 2015). Histopathologically, the characteristic morphologic feature of acute chorioamnionitis is diffuse infiltration of neutrophils of maternal origin into the extraplacental membranes (McNamara et al., 1997; Gravett et al., 2004; Kim et al., 2015).

### Neutrophils and extracellular traps

Colonization of the female genital tract by potential pathogens is a complex process influenced by the host innate immune response and the pathogenic potential of the invading microbe. GBS is distinctive in its ability to cause both invasive disease and asymptomatic colonization of the female genital tract (Goldenberg et al., 2008). GBS is an important pathogen that causes invasive disease in pregnant hosts. In an effort to study how GBS colonizes the reproductive tract and causes ascending invasion of gestational tissues, we employed a mouse model of ascending GBS vaginal infection during pregnancy which was pioneered by Randis et al. (2014). Studies in this model have shown that the GBS toxin beta-hemolysin/cytolysin directly induced disruption of maternal-fetal barriers, causing chorioamnionitis, preterm birth, and stillbirth (Randis et al., 2014). Furthermore, studies of a murine model of chronic GBS genital tract colonization showed significant neutrophil infiltrates in the vaginal mucosa and the formation of DNA neutrophil extracellular traps (NETs) in response to GBS infection (Carey et al., 2014). Human neutrophils have also been shown to form NETs *in vitro* in response to GBS (Carey et al., 2014). NETs are a recently discovered antimicrobial mechanism composed of nuclear chromatin, histones, and other antimicrobial proteins that serve to immobilize and kill or inhibit the growth of invading microbes (Brinkmann et al., 2004).

### Neutrophils and nutritional immunity

High resolution imaging studies have revealed that the DNA which comprises NETs is studded with antimicrobial proteins such as myeloperoxidase, elastin, calprotectin (S100A8/S100A9 heterodimer) and lactoferrin (Brinkmann et al., 2004). The latter two of these host proteins bind transition metals such as manganese, zinc, and/or iron at high affinity to effectively sequester these important nutrients away from invading pathogens in a process referred to as “nutritional immunity” (Becker and Skaar, 2014). Proteomic analyses of amniotic fluid from cases of intra-amniotic infection have revealed dramatic changes in protein composition and increased presence of neutrophil-associated antimicrobial proteins, such as lactoferrin (Gravett et al., 2004; Kim et al., 2015). Previous work indicates that GBS has a strict requirement for iron to initiate bacterial growth in a chemically defined medium (Mickelson, 1966; Willett and Morse, 1966). This, coupled with the observation that lactoferrin is elevated in GBS-infected tissues where neutrophils are abundant (Gravett et al., 2004; Kim et al., 2015), led us to hypothesize that neutrophilic infiltrates, which are characteristically present in chorioamnionitis, could potentially be controlling GBS growth and proliferation in the host by elaborating NETs studded with lactoferrin to chelate nutrient iron and starve invading GBS of essential iron. Our work demonstrates that neutrophils are recruited to the site of GBS infection in a murine model of ascending infection during pregnancy. Furthermore, we demonstrate that GBS-neutrophil interaction results in the elaboration of NETs decorated with the antimicrobial protein lactoferrin. In its apo- (unbound to iron) form, lactoferrin inhibits GBS growth and proliferation in a dose-dependent manner that can be abrogated by the presence of an exogenous source of nutrient metal.

## Materials and methods

### Bacterial strains and culture conditions

The capsular type V *S. agalactiae* strain GB037, obtained from a human case of neonatal sepsis (Davies et al., 2001), was cultured on tryptic soy agar plates supplemented with 5% sheep blood (blood agar plates) at 37°C in ambient air overnight. Bacteria were sub-cultured from blood agar plates into Todd-Hewitt broth (THB) and incubated (aerobically, shaking at 200 RPM) at 37°C in ambient air overnight. The following day, bacterial density was measured spectrophotometrically at an optical density of 600 nm (OD_600_), and bacterial numbers were determined with a coefficient of 1 OD_600_ = 10^9^ CFU/mL.

### Ethics statement

All animal experiments were performed in accordance with the Animal Welfare Act, U.S. federal law, and NIH guidelines. All experiments were carried out under a protocol approved by Vanderbilt University Institutional Animal Care and Use Committee (IACUC; M/14/034), a body that has been accredited by the Association of Assessment and Accreditation of Laboratory Animal Care (AAALAC).

### Mouse model of GBS infection during pregnancy

**Figure 1:**
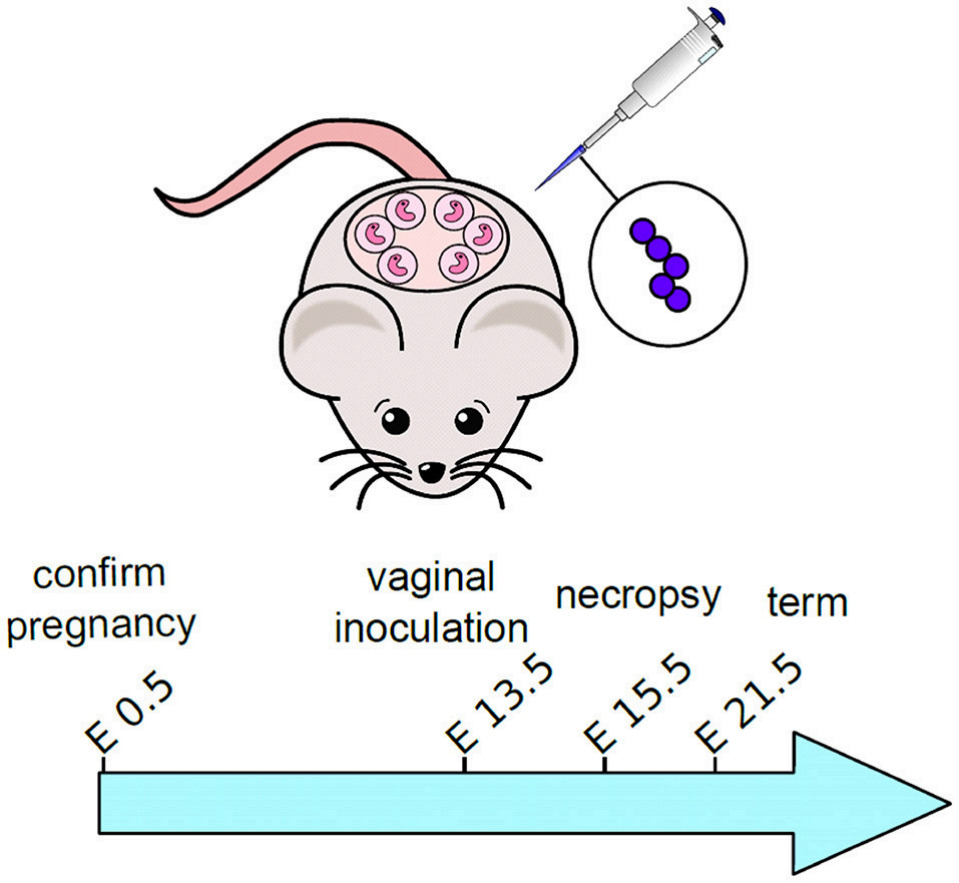
**Schematic representation of experimental design for mouse model of ascending GBS infection during pregnancy**. Pregnant dams were vaginally inoculated with 10^3^ CFU GBS on embryonic day 13.5. Animals were sacrificed and tissues were analyzed 48-h post-infection, on embryonic day 15.5.

GBS infection of pregnant mice and subsequent analyses were performed as previously described with some modifications (Randis et al., 2014). Experimental design is outlined in Figure 1. Briefly, C57BL6/J mice were purchased from Jackson Laboratories and mated in harem breeding strategies overnight. Pregnancy was confirmed the following day by the presence of a mucus plug to establish the embryonic date (E0.5). On embryonic day 13 (E13.5) dams were anesthetized using isoflurane chambers and 50 μL of inocula containing 10^3^ colony forming units (CFU) in THB medium plus 10% gelatin was introduced into the vagina. Sham controls were inoculated with 50 μL of THB medium containing 10% gelatin. Animals were housed singly until embryonic day 15 (E15.5) at which time they were sacrificed by carbon dioxide euthanasia procedures and necropsy was performed to isolate reproductive tissues including vagina, uterus, placenta, decidua, and fetus. For burden studies, a single tissue sample was derived from each separate dam. Four to six animals were utilized for each experimental group derived from three separate experiments (sham treated uninfected animals or GBS-infected animals). Tissues were analyzed for bacterial burden by enumerating CFU per mg of tissue by homogenizing tissues, performing serial dilution, and quantitative culture on blood agar plates.

### Immunohistochemistry and histopathological examination

Murine tissues (1 tissue sample per dam) from the GBS ascending vaginal infection during pregnancy model were fixed in 10% neutral buffered formalin prior to embedment in paraffin. Tissues were cut into 5 μm sections and multiple sections were placed on each slide for analysis. For histopathological examination, sections were stained with hematoxylin and eosin and evaluated by microscopical techniques. Additional sections of fetal-placental tissue were evaluated by immunohistochemical (IHC) techniques to determine the presence and spatial localization of GBS or lactoferrin. After quenching with 0.03% hydrogen peroxide for 20 min at RT, tissues were treated with a heat-induced epitope retrieval solution (Universal decloacker, Biocare Medical, Concord, CA) using a pressure cooker at 121°C for 20 min and allowed to cool at RT prior to blocking with 10% normal goat serum in PBS pH 7.4 0.1M. Primary antibody, either a rabbit polyclonal antibody to GBS (Abcam, ab78846) or a mouse monoclonal antibody to lactoferrin (Abcam, ab166803), was applied for 1 h. Detection of primary antibody was performed using the HRP-Polymer system for 30 min and developed with 3, 3′-diaminobenzidine tetrahydrochloride (DAB) (Dako, Carpinteria, CA). The sections were counterstained with hematoxylin, rinsed, dehydrated, and mounted with Cytoseal XYL before light microscopy analysis was performed. Micrograph images were analyzed by ImageJ IHC toolbox plugin to quantify H-DAB staining by color detection, converted to 8-bit format and densitometry quantification as previously described (Haley et al., 2015).

### Flow cytometry evaluation of neutrophils

Flow cytometry analyses were performed as previously described with some modifications (Shynlova et al., 2013). Four to seven placenta and decidua were collected from each animal (4–6 animals total) and pooled, weighed, minced with sterile scissors and digested in a solution containing 1 mg/mL collagenase, 1 mg/mL hyaluronidase, and 150 μg/mL DNAse I with agitation for 1 h at 37°C. A total of 40 mL of digestion solution was used per sample (each sample consisted of a single animal's pooled placenta or decidua, totaling 4–7 per animal) and samples were washed using RPMI +/+ medium (containing 1% antibiotic and 10% fetal bovine serum), centrifuged at 1500 RPM at 4°C for 10 min followed by 100 μm nylon mesh filtration to eliminate remaining particulates. Cells were then washed, as previously described, and the filtrate was resuspended in 25% Percoll in RPMI +/+, overlaid onto 50% Percoll, with 2 mL PBS layered above the 25% Percoll. Percoll gradient was centrifuged for 35 min at 1500 RPM without the brake and cells were washed as before, followed by 10 min at room temperature in RBC lysis buffer (eBiosciences). After two more washes with RPMI +/+, cells were counted and one million cells were aliquoted into flow cytometry tubes. Cells were surface stained for 20 min at 4°C with anti-mouse CD45, anti-mouse CD11b, anti-mouse Neu7/4 (Ly-6B.2), anti-mouse GR1 (1A8-Ly6g), and FcR blocking reagent. Isotype controls were included in separate tubes. After surface staining, all cells were washed with PBS and stained with a live/dead viability dye (Life Technologies) for 30 min at 4°C. Cells were then washed with 4 mL PBS containing 1% BSA (FACS buffer), fixed for 15 min at 4°C with 1% paraformaldehyde in PBS, and washed again with FACS buffer. Cells were resuspended in FACS buffer and immediately acquired on a BD LSR2 Flow Cytometer (BD Biosciences). Analysis was performed with BD FACS Diva software (BD Biosciences).

### Elicitation and isolation of murine neutrophils

Murine neutrophils were prepared by peritoneal casein elicitation protocol as previously described (Swamydas et al., 2015). Briefly, C57BL6/J mice (both male and female) aged 6–10 weeks old were purchased from Jackson Laboratories for these experiments. Mice were anesthetized with isofluorane and 1 mL of sterile casein solution (9% w/v in 1X PBS pH 7.2 containing 0.9 mM CaCl_2_ and 0.5 mM MgCl_2_) was injected into the peritoneal cavity of each mouse (2–4 mice per experiment). The following day, a second casein injection was performed and 4 h after the second injection, mice were sacrificed and neutrophils were isolated from the peritoneal cavity in 3–5 mL of sterile PBS. Cells were collected and washed in 1X red blood cell lysis buffer (RBC Lysis Buffer, Sigma Aldrich) before placing in RPMI 1640 medium supplemented with 10% fetal bovine serum, L-glutamine, and HEPES buffer. Validation of cell purity by flow cytometry techniques described above reveals this protocol routinely yields >95% pure neutrophil populations.

### Confocal laser scanning microscopy

Neutrophils isolated as described above were placed onto 12 mm poly-L-lysine coated coverslips (ThermoFisher) and allowed to adhere to the surface for 1 h before inoculation with bacterial cells. Bacteria were added to neutrophils at a multiplicity of infection of 50:1 and co-cultured for 24 h in the presence or absence of 1 μg/mL of DNAse. Concomitantly, uninfected samples were maintained as negative controls. Cells were stained with 1 μM Sytox green (green, Life Technologies) and 30 nM 4′,6-diamidino-2-phenylindole (DAPI, blue, Life Technologies) to target DNA, and mouse monoclonal antibody to lactoferrin (Abcam, ab166803) plus secondary goat-anti-mouse antibody conjugated to Alexa Fluor 647 (red) were applied to cells, agitating for 1 h. Cells were washed with phosphate buffered saline (pH 7.4) three times before mounting onto slides and visualizing with a Zeiss LSM 710. Sytox green is an impermeable dye which is used to stain extracellular traps and is also used to differentiate between live and dead cells (Brinkmann et al., 2010). DAPI stains condensed chromatin bright blue with increased intensity proportional to chromatin condensation, making it an efficient nuclear stain (Mascetti et al., 2001). When used in combination, Sytox green will stain extracellular DNA bright green, while DAPI stains nuclear chromatin blue. However, in the case of samples evaluated for NETosis where neutrophils frequently undergo cell death during the process, Sytox green enters dying cells staining both the nucleus, and the extracellular fibers green (resulting in co-staining of the nucleus both blue and green). NETs were identified by Sytox green staining of fibers extending outside of the cell nucleus containing condensed chromatin (DAPI-positive, blue regions). Images were analyzed in both widefield and confocal modalities at 630X magnification and micrographs were collected with Zen 2010 software. Micrographs shown are representative of three biological replicates. Images were analyzed and statistical analyses were performed with ImageJ and GraphPad Prism software. A total of 151–192 cells were quantified for each condition derived from more than 12 fields.

### Field-emission gun scanning electron microscopy

Murine neutrophils isolated from the co-culture procedure described above were prepared for scanning electron microscopy analyses as previously described (Brinkmann et al., 2010). Samples were subjected to washing three times with 0.05 M sodium cacodylate buffer and fixing in 2.0% paraformaldehyde, 2.5% gluteraldehyde in 0.05 M sodium cacodylate buffer for 4 h. Secondary fixation with 0.1% osmium tetroxide was performed for 15 min prior to sequential dehydration with increasing concentrations of ethanol. Samples were dried at the critical point using a CO_2_ drier (Tousimis), mounted onto an aluminum stub, and sputter-coated with 80/20 gold-palladium. A thin strip of colloidal silver was painted at the sample edge to dissipate sample charging. Samples were imaged with an FEI Quanta 250 field-emission gun scanning electron microscope (FEG-SEM). Images are representative of three replicates from three different experiments.

### Preparation of holo- or apo-lactoferrin

Iron-bound (holo-) or unbound (apo-) lactoferrin was prepared as previously described (Senkovich et al., 2010). Briefly, 10 mM stock of lactoferrin (Sigma Aldrich) was dialyzed against either 0.1 M sodium citrate-bicarbonate buffer pH 8.2 alone to generate apo-lactoferrin, or buffer containing 70 mM ferric chloride to generate holo-lactoferrin. Both apo- and holo-lactoferrin were dialyzed against 1X phosphate buffered saline (PBS) containing Chelex Resin (Sigma Aldrich) to remove any unbound iron content.

### Bacterial growth and viability analyses

For growth and viability analyses, bacteria were cultured overnight in THB (to an OD_600_ of ~0.8), then sub-cultured by performing a 1:100 dilution (roughly 8 × 10^6^ CFU/mL) in 60% THB plus 40% calprotectin buffer (100 mM NaCl, 3 mM CaCl_2_, 20 mM Tris pH 7.5; Senkovich et al., 2010; Haley et al., 2015) referred to as “medium alone” or medium supplemented with increasing concentrations (100, 250, 500, 750, 1000 μg/mL) of purified apo- or holo-lactoferrin protein alone or supplemented with 100 μM ferric chloride. These concentrations span the physiological range at which lactoferrin would be present in inflamed tissues and neutrophil granules (Masson et al., 1969; Guillen et al., 1998; Simard et al., 2011). Additionally, bacteria were grown in increasing concentrations of the synthetic chelator 2, 2′-dipyridyl (25, 50, 100, 150, 200, 250, 300, 350, 400 μM) in medium alone or medium supplemented with 250 μM ferric chloride. Bacterial growth was evaluated at 24 h post-inoculation by spectrophotometric reading of OD_600_ or bacterial viability was evaluated at 24 h by serial dilution and plating onto blood agar plates and quantifying viable colony forming units per mL of culture (CFU/mL).

### Statistical analyses

Statistical analysis of flow cytometry and neutrophil NET quantifications was performed using Student's *t*-test and One-Way ANOVA, respectively. Bacterial growth assays were analyzed by One-Way ANOVA with either Tukey's or Dunnet's *post-hoc* multiple correction tests and Student's *t*-test. *P* ≤ 0.05 were considered significant. All data analyzed in this work were derived from at least three separate biological replicates. Statistical analyses were performed using GraphPad Prism Software (Version 6.0, GraphPad Software Inc., La Jolla CA) and Microsoft Excel (Version 14.6.3, Microsoft Corporation, Redmond WA).

## Results

### GBS invades gestational tissues to cause invasive ascending infection during pregnancy

**Figure 2:**
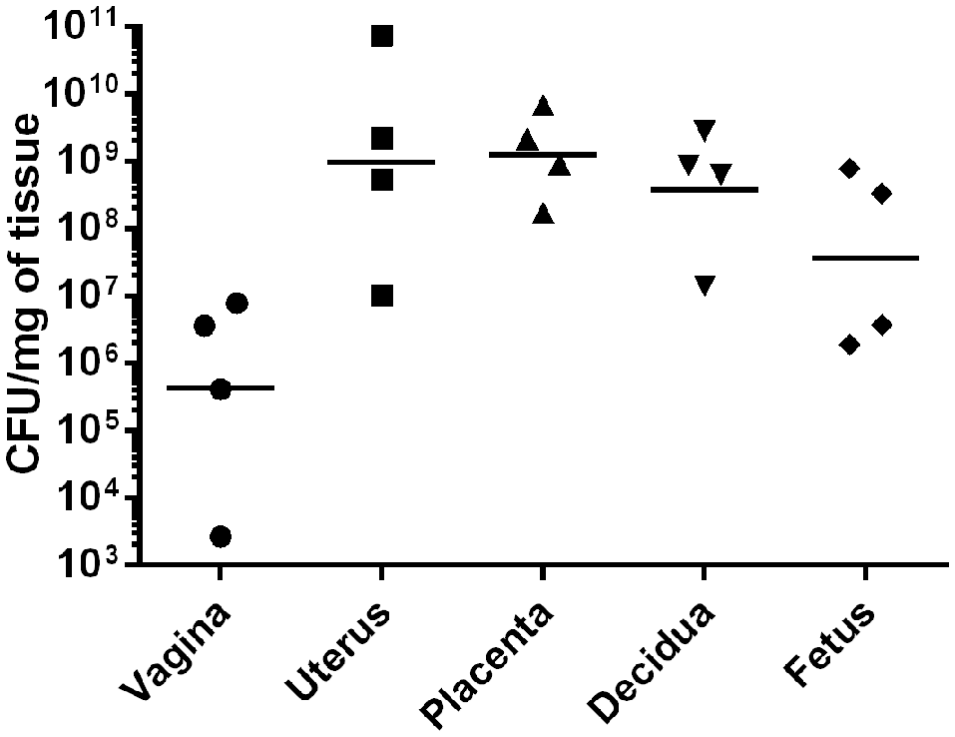
**GBS invades reproductive tissues and disseminates in the gravid host**. Quantitative culture from tissues harvested from pregnant mice infected with GBS reveals bacterial presence in numerous gestational tissues. GBS bacteria ascend from the vagina to the uterus and invade the decidua, placenta, and fetus (*N* = 4). Points represent burden value from 1 tissue derived from 1 dam. Horizontal lines represent the geometric mean of bacterial burden.

To evaluate GBS ascending infection during pregnancy, we utilized the mouse model of ascending GBS vaginal infection as previously described (Randis et al., 2014) and developed new outputs from this model by employing bacteriological culturing and IHC microscopy assays to evaluate bacterial burden in discrete tissue compartments within the gestational tissue. Our results reveal that GBS colonizes surface of the vagina (Supplemental Figure 1, Figure 2) at an average burden of 3.6 × 10^6^ CFU/mg of tissue, and the bacterial colonization is spatially oriented at the lumen of the tissue within the vaginal mucosa as determined by IHC microscopic examination (Supplemental Figure 1). Furthermore, IHC microscopic analyses reveal GBS ascends in this model and colonizes at the lumen of the endometrium within the uterus, averaging 2.2 × 10^9^ CFU/mg (Supplemental Figure 2). IHC analyses also reveal that GBS invades the decidua and the placenta, and quantitative culture demonstrates bacterial burden averages 8.5 × 10^8^ CFU/mg in the decidua, 2.1 × 10^9^ CFU/mg in the placenta, and 3.3 × 10^8^ CFU/mg in the fetus.

### GBS infection results in profound inflammation including recruitment of neutrophils to the placenta and the choriodecidua

**Figure 3:**
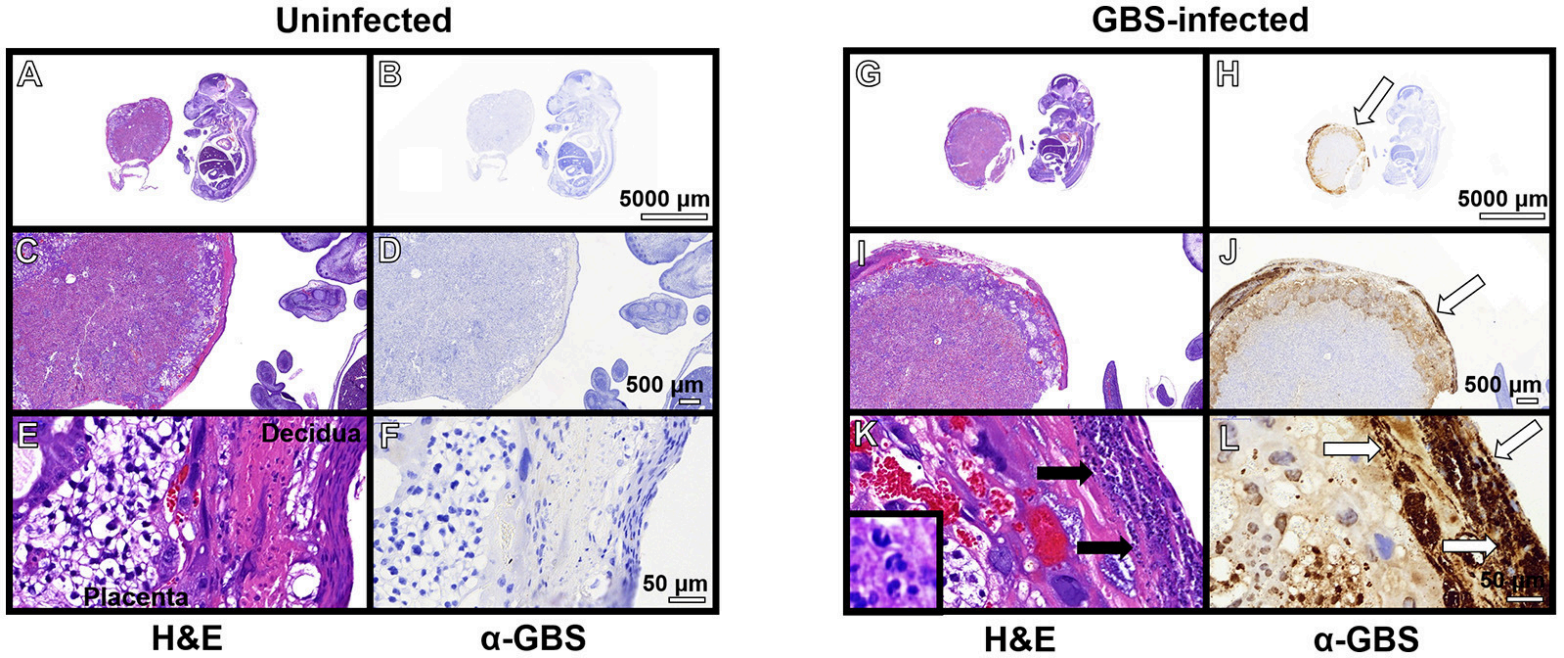
**Immunopathological analyses of fetal-placental units**. Hematoxylin and eosin (H&E) stained samples of uninfected **(A–F)** and GBS-infected **(G–L)** fetal-placental units reveal profound infiltration of polymorphonuclear cells in response to infection (**K**, black arrows), a result that was not observed in uninfected animals **(E)**. IHC analyses using a polyclonal rabbit antibody to total GBS cell lysate (α-GBS) indicate GBS penetrates the decidua and the placenta to invade the gestational tissues (**H,J,L**, white arrows), a result that was not seen in uninfected animals **(B,D,F)**. GBS staining is found within tissue compartments containing polymorphonuclear cell infiltrates indicating neutrophils are present at the site of infection (representative images of *N* = 3–5).

**Figure 4:**
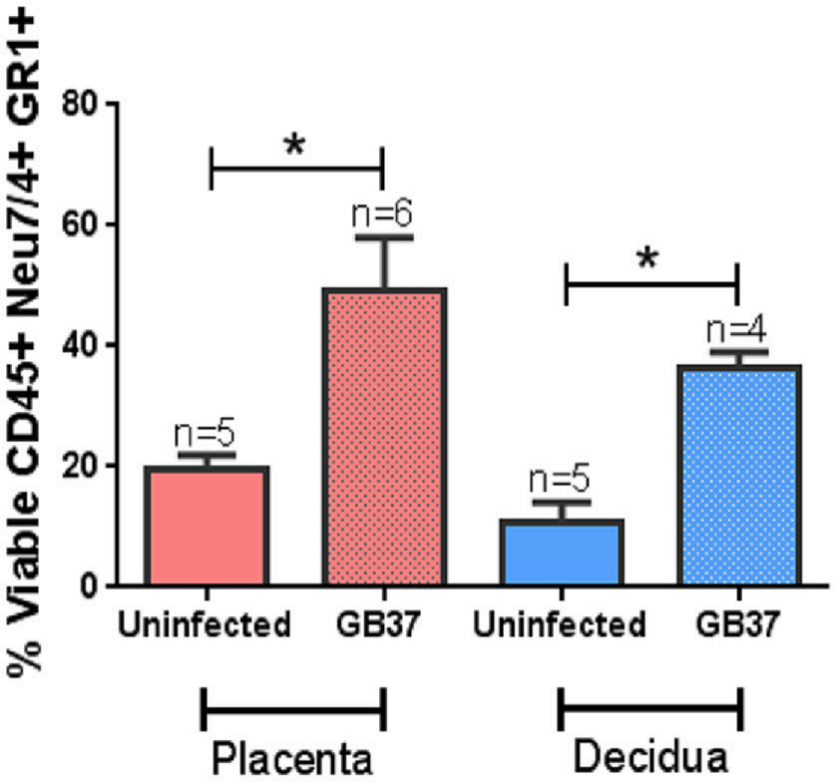
**Flow cytometry analyses of placenta and decidua to evaluate the relative abundance of neutrophils**. Neutrophils were evaluated by CD45+ Neu7/4+ GR1+ gating strategy on cells derived from the placental or decidual compartment of uninfected or GBS-infected pregnant mice. Bars represent mean percent (viable cells CD45+ Neu7/4+ GR1+) ± SEM. GBS-infection results in enhanced presence of neutrophils in the decidua and placenta (^*^P < 0.05 Student's *t*-test, *N* = 4–6).

Previous work indicates the murine model of ascending GBS infection during pregnancy mirrors the human histopathological changes associated with chorioamnionitis (McNamara et al., 1997). In an effort to better characterize the inflammation present within this model, we employed the immunological tools readily available for utilization with this mouse model and performed both histopathological examination and correlative flow cytometry analyses using antibodies to CD45, Neu7/4, and GR1 which are specific for murine neutrophils (Supplemental Figure 3) and CD45+/Neu7/4+/GR1+ gating strategies. Histopathological examination reveals profound infiltration of polymorphonuclear cells within GBS-infected decidua and placenta, a result that was not seen in uninfected samples (Figure 3). This result was recapitulated using flow cytometry to analyze the placenta and decidua compartments, demonstrating 54% of viable CD45+ cells were Neu7/4+/GR1+ neutrophils in GBS-infected placenta vs. 19% of uninfected placenta, a significant increase in neutrophils infiltrating the placenta in response to GBS infection (*P* < 0.05). Similarly, 61% of viable CD45+ cells were Neu7/4+/GR1+ neutrophils in GBS-infected decidua vs. 16% of uninfected decidua, a significant increase in neutrophils in the decidual compartment in response to GBS infection (Figure 4, *P* < 0.05). Furthermore, the preponderance of PMNs discovered in the decidual and placental compartment is associated with GBS staining, indicating neutrophils were recruited to the site of GBS infection within each tissue compartment.

### Lactoferrin is associated with neutrophils in the decidua and placenta in response to GBS infection

**Figure 5:**
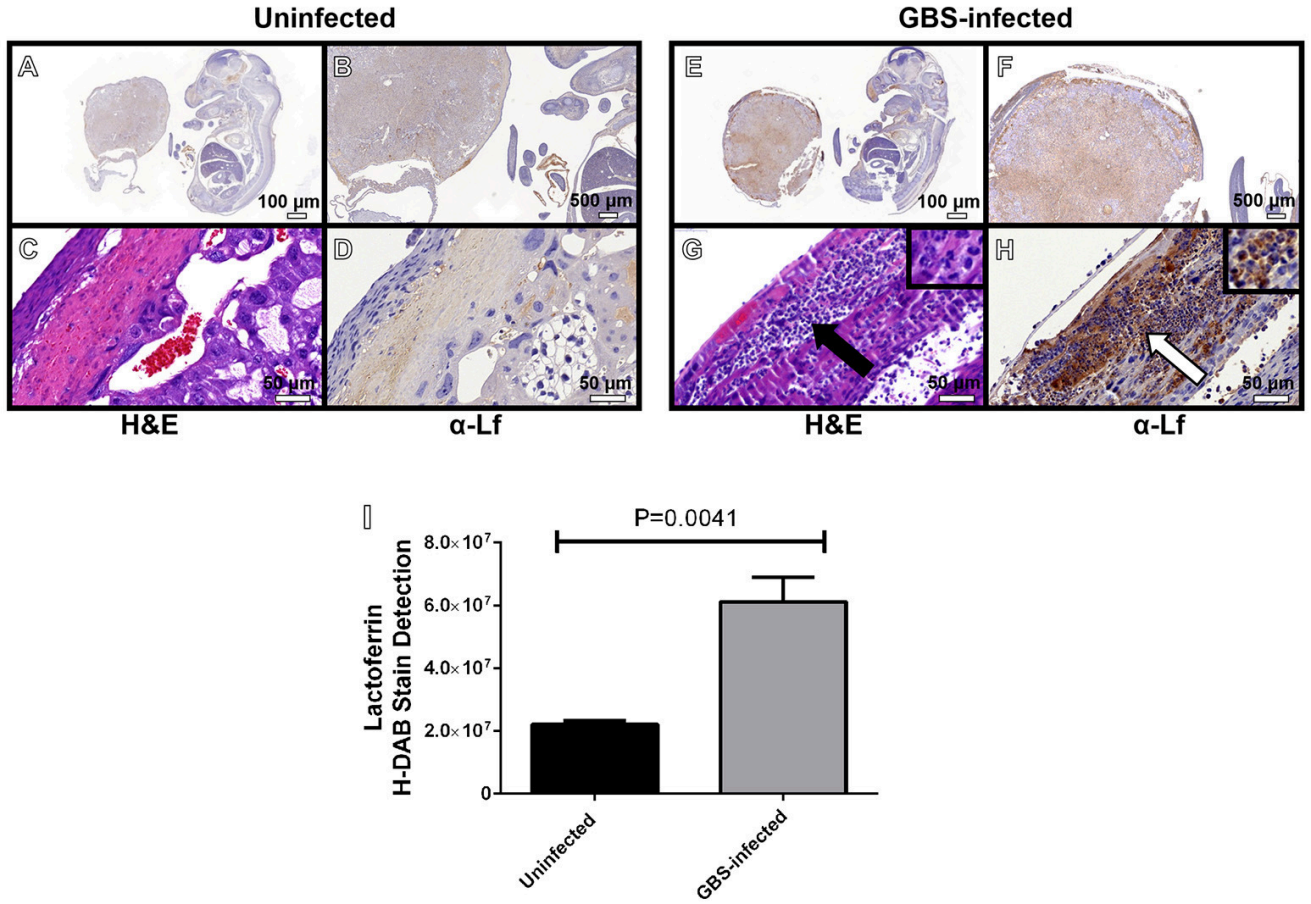
**Microscopic analyses of the spatial localization of lactoferrin within uninfected (A–D)** or GBS-infected **(E–H)** fetal-placental tissue. Representative micrographs collected at 4X **(A,E)**, 20X **(B,F)**, and 400X **(C,D,G–H)**. Panels **(A,B,D,E,F,H)** indicate IHC staining with a mouse monoclonal antibody to lactoferrin (α-Lf). Panels **(C,G)** indicate hematoxylin and eosin staining (H&E) for immunopathology. Panel **(I)** quantification of anti-lactoferrin HDAB stain by ImageJ IHC toolbox reveals GBS infection results in elevated lactoferrin within the fetal-placental tissue (bars indicate mean quantified H-DAB stain ± SEM, *P* = 0.0041, Student's *t*-test, *N* = 3). Panels **(G–H)** demonstrate the preponderance of lactoferrin stain (white arrow) is associated with polymorphonuclear cells (black arrow).

Neutrophils play an important role in innate immunity against bacterial pathogens. One way they accomplish this is by secreting molecules with antimicrobial activity. Previous work has shown that lactoferrin is elevated in patients with intra-amniotic infections during pregnancy, disease states that are often associated with high proportions of neutrophilic infiltration of both the decidua and the placenta (McNamara et al., 1997; Gravett et al., 2004; Kim et al., 2015). We hypothesized that neutrophils recruited to the site of GBS infection could produce lactoferrin as an antimicrobial strategy. To test this, IHC analyses were employed to determine the spatial localization of lactoferrin within GBS-infected or uninfected gestational tissues (Figure 5). Lactoferrin was highly abundant within the decidua and placenta of GBS-infected pregnant mice, a result that was not observed in uninfected control animals. Interestingly, lactoferrin staining was largely associated with areas of tissue exhibiting profound neutrophilic infiltrates (Figures 5G,H), and tissue compartments that stain positive by IHC for GBS in, indicating this protein could be associated with neutrophils which are found at the site of GBS infection.

### Neutrophils encountering GBS produce extracellular traps comprised of DNA

**Figure 6:**
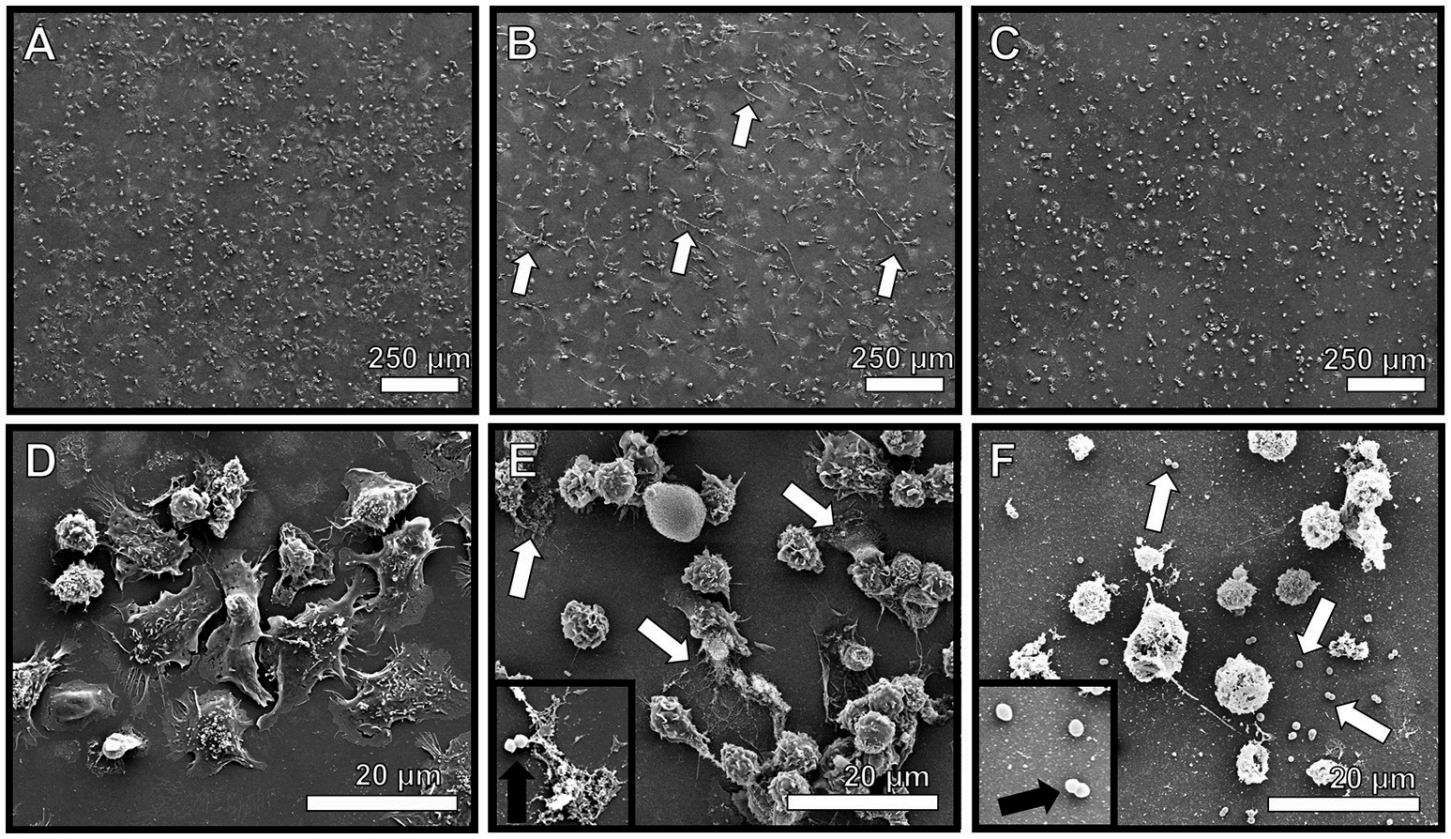
**Neutrophils encountering GBS elaborate extracellular traps (NETs) comprised of extracellular DNA**. High resolution scanning electron microscopy analyses reveals murine neutrophils elaborate extracellular traps in response to GBS co-culture (**B,E**, white arrows), a result which was not seen in uninfected mouse neutrophils **(A,D)**. Treatment with DNAse I abrogates observation of extracellular traps (**C,F**, white arrows), indicating traps are structurally comprised of DNA. Inset panels of **(E,F)** show GBS associated with NET fibers or not associated with NETs due to DNA degradation, respectively (black arrows).

**Figure 7:**
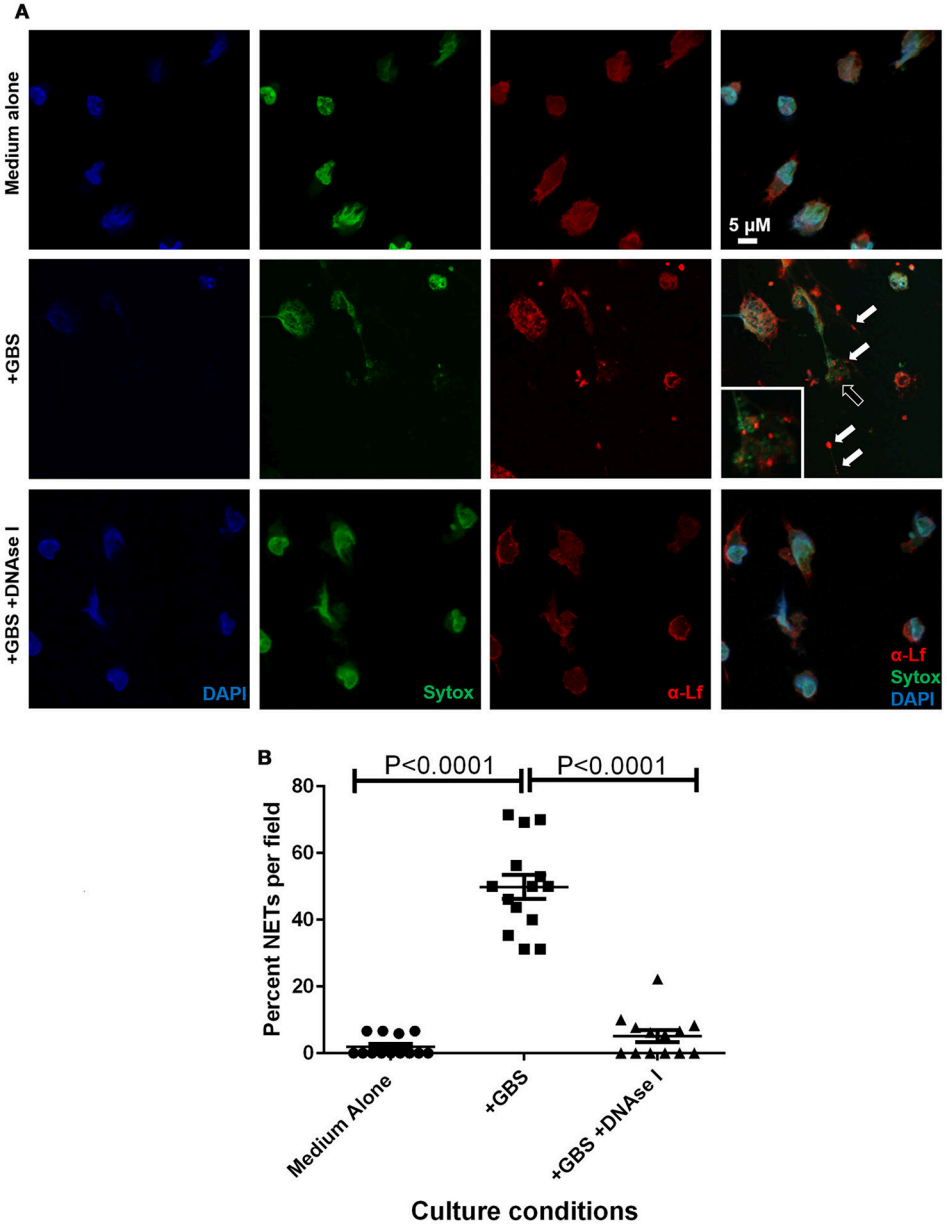
**Extracellular traps formed in response to GBS are decorated with lactoferrin**. Panel **(A)** Confocal laser scanning microscopy analyses of neutrophils cultured in the absence of GBS (Medium Alone), in the presence of GBS (+GBS) alone or in co-cultures supplemented with DNAse I (+GBS +DNAse I) were subjected to intracellular nuclear DNA stain (DAPI, blue), extracellular DNA stain (Sytox, green), or antibody to lactoferrin (α-Lf). Merge images reveal that GBS co-culture with neutrophils results in the elaboration of neutrophil extracellular traps comprised of extracellular DNA (representative images of *N* = 3 biological replicates). These traps immobilize GBS (black arrow), and are studded with lactoferrin (white arrow) which is found in close contact with GBS, indicating lactoferrin is part of the antimicrobial repertoire secreted in the NETs in response to GBS. Panel **(B)** Enumeration of the percentage of NETs per field was performed, and demonstrate that NETs are enriched in GBS-containing co-cultures, but are ablated by treatment with DNAse I (Bars indicate mean percent of NETs per field ± SEM, *P* < 0.0001, One Way ANOVA, *N* = 3 biological replicates and at least five fields per replicate, 150+ cells total).

Due to the observation that GBS staining frequently co-localized with PMN infiltrates, we hypothesized that GBS-neutrophil interactions could influence the outcome of disease progression. Specifically, it was hypothesized that neutrophils exert an antimicrobial activity against GBS by secreting antimicrobial molecules, such as lactoferrin. To further evaluate this activity, *ex vivo* isolation of murine neutrophils was performed and these cells were co-cultured with GBS at an MOI of 50:1 bacterial to host cells. Co-cultures were evaluated by high resolution microscopical techniques including high resolution field-emission gun scanning electron microscopy (FEG-SEM) and confocal laser scanning microscopy (CLSM). FEG-SEM results in Figure 6 indicate that neutrophil exposure to GBS results in enhanced neutrophil extracellular trap formation. Similarly, confocal analyses reveal GBS-treated samples have 24.5-fold enhanced neutrophil extracellular trap (NET) formation compared to uninfected controls (Figure 7, *P* < 0.05). These extracellular traps were ablated by treatment of co-cultures with DNAse I, confirming that DNA is a major component of these structures (Figures 6, 7). Furthermore, CLSM analyses in Figure 7 in which co-cultures were stained with Sytox Green (a non-permeant DNA stain, green) as well as DAPI (condensed chromatin DNA stain, blue) indicated extracellular traps, which appear to have GBS caught within their margins, are staining Sytox Green positive, confirming these are comprised of extracellular DNA. Conversely, uninfected controls and DNAse I-treated GBS-neutrophil co-cultures have fewer apparent extracellular traps staining with Sytox Green fibers outside of the cell nucleus (*P* < 0.05).

### Neutrophil extracellular traps comprised of DNA are studded with the antimicrobial glycoprotein lactoferrin

Previously published work indicates that lactoferrin secretion is enhanced in gestational tissues in response to GBS infection (Gravett et al., 2004), and that lactoferrin is a putative antimicrobial protein which decorates the NET (Brinkmann et al., 2004). We hypothesized that NET formation in response to GBS exposure could result in secretion of lactoferrin into the NET. To test this, we employed CLSM and immunofluorescence approaches to analyze subcellular spatial localization of lactoferrin (Figure 7, anti-lactoferrin and Alexa Fluor 647 conjugated secondary antibody, red) with respect to the extracellular trap (Sytox Green, green). Our results indicate lactoferrin secretion into the extracellular trap was induced by GBS co-culture with PMNs. The lactoferrin associated with extracellular DNA that comprised the NET, and was found spatially localized near structures consistent in size and arrangement with GBS cells, indicating lactoferrin may interact with GBS within the NET. DNAse I treatment of the co-culture resulted in diminished NETs observed by CLSM, and this treatment also resulted in decreased extracellular lactoferrin and GBS-associated with PMNs, indicating lactoferrin was associated with the DNA which structurally comprises the extracellular trap, and that the NETs potentially served to immobilize GBS and expose them to antimicrobial insults including lactoferrin.

### Lactoferrin exerts antimicrobial activity against GBS via iron sequestration

**Figure 8:**
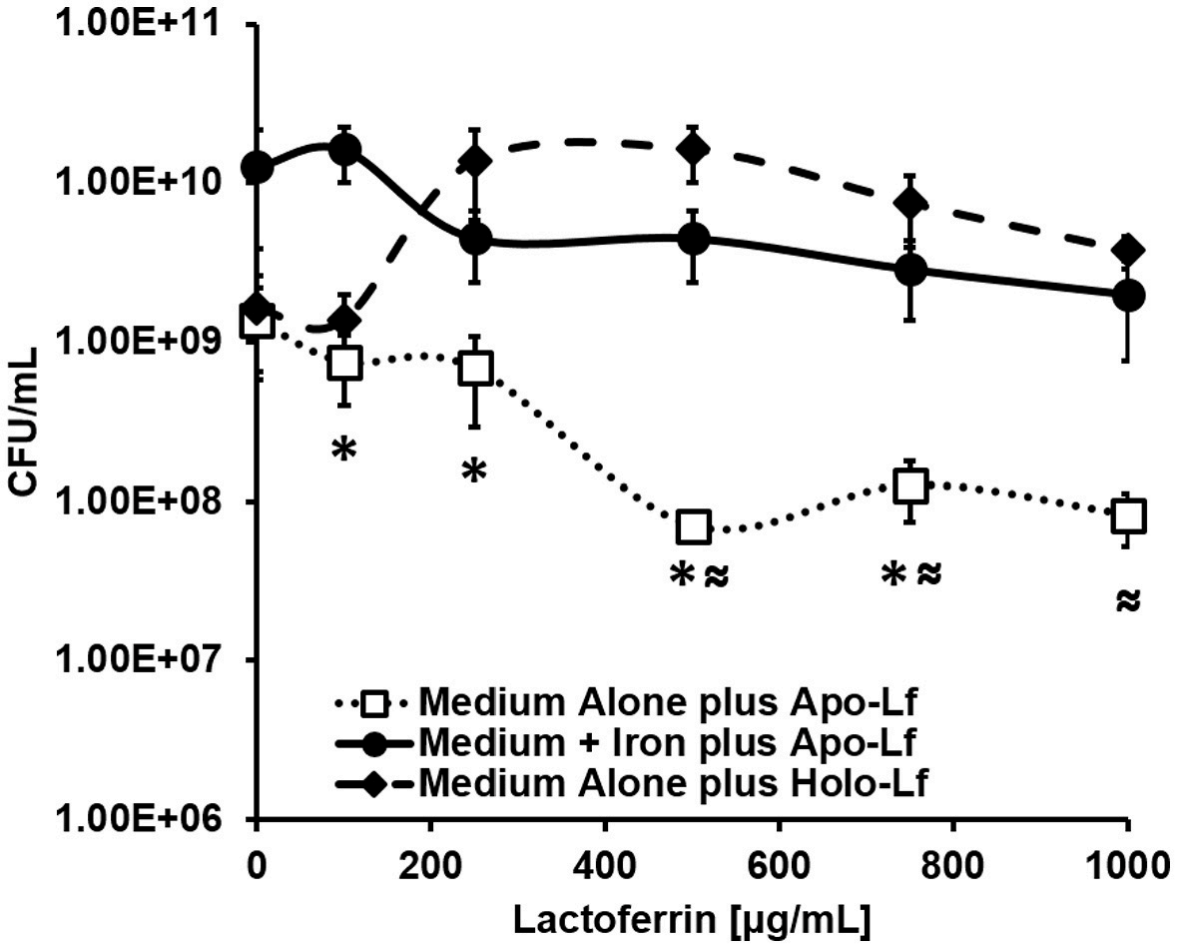
**Analysis of bacterial viability after 24 h of culture**. Exposure to apo-lactoferrin (Apo-Lf, open squares) results in decreased GBS growth and viability in a dose-dependent manner, a result which was reversed by the addition of a source of exogenous nutrient iron (closed circles). Conversely, exposure to increasing concentrations of holo-lactoferrin (Holo-Lf, closed diamonds) does not significantly inhibit bacterial viability. Points indicate mean CFU/mL ± SEM per timepoint (*N* = 3 biological replicates). ^*^P < 0.05, compared to Medium + Iron plus Apo-Lf, ^≈^*P* < 0.05, compared to Medium plus Holo-Lf.

Because lactoferrin is an antimicrobial glycoprotein that binds and sequesters iron in a strategy to chelate nutrient iron away from invading microorganisms, we hypothesized that the deposition of lactoferrin within neutrophil extracellular traps could potentially inhibit GBS growth and viability. To test this, bacterial growth and viability assays were performed. Results in Figure 8 indicate that at concentrations of 100, 250, 500, 750, and 1000 μg/mL, apo-lactoferrin represses GBS growth 46, 50, 95, 91, and 94%, respectively, compared to cells cultured in medium alone. This result was recapitulated using the synthetic iron chelator 2, 2′ dipyridyl which has antimicrobial activity against GBS (Supplemental Figure 4). Interestingly, supplementation with 100 μM ferric chloride as an exogenous source of nutrient iron restored bacterial cell density to levels that were comparable or higher than those grown in medium alone (Figure 8, Supplemental Figure 4). Results in Figure 8 indicated that even at concentrations as high as 1000 μg/mL, holo-lactoferrin did not repress bacterial growth significantly compared to medium alone, a result that could be attributed to the fact that the iron-loaded form of this protein did not exert iron chelation activity on the bacteria within this particular growth medium. Taken together, these results indicate that the iron-sequestration activity of apo-lactoferrin has detrimental effects on GBS; diminishing both bacterial growth and viability through micronutrient starvation.

## Discussion

### GBS invades reproductive tissues, crosses the placenta, and disseminates to the fetus

Colonization of the vaginal mucosa is a critical primary step in GBS pathogenesis during pregnancy. Once colonized, bacteria ascend into the intrauterine cavity, transverse the fetal membranes, and cause severe neonatal disease outcomes including preterm birth, neonatal sepsis, and neonatal demise. The advent of a model of ascending GBS infection in a pregnant mouse provides numerous genetic and immunological tools to study the complex dialogue between host and pathogen during pregnancy (Randis et al., 2014). We have further refined this model by dissecting the gestational tissue compartments and analyzing both enumeration of GBS burden and spatial distribution of bacteria by quantitative culture and immunohistochemical techniques. These experiments reveal that bacterial invasion could be observed in the vagina, uterus, decidua, placenta, and fetus with the highest burden being present in the uterus and the burden titrating as the bacteria penetrate the gravid host, as is expected in a model of invasion.

### The murine model of ascending vaginal infection by GBS results in chorioamnionitis which is characterized by a profound infiltration of PMNs to the choriodecidua and placenta

In response to GBS infection, pro-inflammatory signaling cascades are promoted which ultimately lead to the recruitment of innate immune cells to the site of infection (Randis et al., 2014). Concordant with this, our work demonstrates that GBS infection elicits significant PMN recruitment to the placenta (2.5-fold increase compared to uninfected controls) and the decidua (3.3-fold increase compared to uninfected controls), a result that is in agreement with experiments performed in previous studies using this model indicating inflammation-related pathology scores are elevated in GBS-infected animals compared to sham-treated controls (Randis et al., 2014). It is interesting to note that the neutrophilic infiltrate within the gestational tissues mirrors the clinical presentation of chorioamnionitis in human subjects (McNamara et al., 1997; Gravett et al., 2004; Goldenberg et al., 2008). This neutrophil response likely initiates an antimicrobial response, through phagocytosis and bacterial killing, as well as through the deposition of antimicrobial molecules at the site of infection. Furthermore, it is likely that neutrophil presence results in the stimulation of pro-inflammatory signaling cascades which initiates further inflammation pathways leading to perturbed maternal-fetal tolerance and tissue destruction (Marzano et al., 2010; Simard et al., 2011; PrabhuDas et al., 2015; Tsatsaronis et al., 2015).

### Murine PMNs form NETs in response to GBS

Neutrophils play a critical role in controlling invading pathogens (Kobayashi and DeLeo, 2009). Emerging evidence indicates that one pathway neutrophils employ to combat pathogenic microbes is elaboration of NETs (Brinkmann et al., 2004). NETs function as a structural component of the innate immune response which serves to immobilize microorganisms (Brinkmann et al., 2004). NETs are largely comprised of DNA and associated histones which are decorated with antimicrobial molecules (Brinkmann et al., 2004; Urban et al., 2006). Because NETs can immobilize bacteria, bringing them into intimate contact with these antimicrobial proteins and glycoproteins, it is purported that NETs are an important arm of the antimicrobial defense strategies employed by the host (Brinkmann et al., 2004; Urban et al., 2006).

Our work indicates murine neutrophils deploy extracellular traps in response to encountering GBS. Previous work has shown that GBS-infected human tissues have abundant neutrophilic infiltrates and GBS induces NETosis in human neutrophils as determined by immunohistochemical and microscopical analyses using neutrophil elastase and histones, markers which are commonly associated with NETs (Carlin et al., 2009; Derré-Bobillot et al., 2013; Yan et al., 2013; Moon et al., 2014; Okumura and Nizet, 2014; Xu et al., 2015). Interestingly, work by Carey et al. shows that induction of NETosis is hemolysin-dependent, indicating that GBS virulence factors participate in this process (Carey et al., 2014). Derre-Bobillot et al., report that GBS encodes a DNAse which is critical for liberating the bacterial cell from the murine NET, indicating GBS has mechanisms to evade this immunological response (Derré-Bobillot et al., 2013). It is worthy to note that in this report, the authors utilized murine PMNs which were elicited by thioglycollate treatment and subsequently stimulated with PMA (Phorbol-12-Myristate-13-Acetate) to induce murine NET formation. Murine NETs induced during co-culture with GBS are comprised of extracellular DNA, a result which agrees with previously published reports (Brinkmann et al., 2004; Carey et al., 2014). Our approach to studying GBS-dependent induction of NET formation has been employed by other groups utilizing extracellular DNA stains such as Sytox Green, or the biochemical approach of DNAse-dependent degradation of NETs to determine the association of extracellular DNA with NET structures (Carlin et al., 2009; Yan et al., 2013).

### NETs induced by GBS contain lactoferrin

It is increasingly appreciated that NETs are reservoirs of antimicrobial molecules (Brinkmann et al., 2004; Jean et al., 2016). A wide repertoire of antimicrobial molecules including calprotectin, lactoferrin, and cathelicidin has been associated with NETs (Bennike et al., 2015). Many of these have been identified by unbiased proteomics techniques, targeted immunohistochemical techniques, and microbiological killing assays. These antimicrobial molecules are important mediators of innate immunity. Interestingly, both calprotectin and lactoferrin participate in chelation of nutrient metals, a process that effectively sequesters transition metals away from pathogenic microbes (Becker and Skaar, 2014; Gaddy et al., 2014). Nutritional immunity is progressively recognized as a critical way to combat microbial infection (Becker and Skaar, 2014; Gaddy et al., 2014; Haley et al., 2015). Lactoferrin is of particular interest as GBS induces elevated lactoferrin levels during infection of placental membranes, however the cellular contribution for this was obscure (Boldenow et al., 2013). Furthermore, elevated lactoferrin levels have been associated with GBS infection in neonates such as septicemia (Gutteberg et al., 1990, 1991). We report that GBS-induced NETs are a source of lactoferrin, however the role of these structures and antimicrobial molecules in the context of infection during pregnancy remains unknown.

### Lactoferrin has antimicrobial activities against GBS

Lactoferrin is a neutrophil-associated glycoprotein with immunomodulatory properties which has long been recognized as a broad-spectrum antimicrobial agent against numerous microorganisms (Arciola, 2010; Savchenko et al., 2011; Scapinello et al., 2011; Svobodová et al., 2012; Barrientos et al., 2013; Björnsdottir et al., 2016; Carretta et al., 2016; Feintuch et al., 2016; Okubo et al., 2016; van der Spek et al., 2016). This activity is associated with lactoferrin's intrinsic iron-binding properties which participate in nutritional immunity via iron sequestration (Weinberg, 1975, 1977; Bezkorovainy, 1981; Ellison, 1994). Iron is a strict nutritional requirement for numerous bacteria including GBS, thus chelation of this critical molecule could alter bacterial cell biology (Mickelson, 1966; Willett and Morse, 1966). Our results indicate that apo-lactoferrin at 100, 250, 500, 750, 1000 ug/mL resulted in 1.8, 2.0, 19.6, 10.9, 16.7-fold, decreased bacterial viability, respectively. Work by other groups indicates bovine lactoferrin has been demonstrated to have bacteriostatic activity against GBS, and human lactoferrin has been observed to bind to lipoteichoic acids in *Streptococcus* spp., inhibit biofilm formation, and activate the complement pathway, indicating it has immunomodulatory and pleotropic effects (Rainard, 1986, 1993; Berlutti et al., 2004; Andréa et al., 2015). Interestingly, lactoferrin administration as a prebiotic is under consideration for women with preterm delivery and lactoferrin supplementation to formula has been shown to limit GBS growth >50% (Otsuki et al., 2014; Trend et al., 2015).

## Conclusions

**Figure 9:**
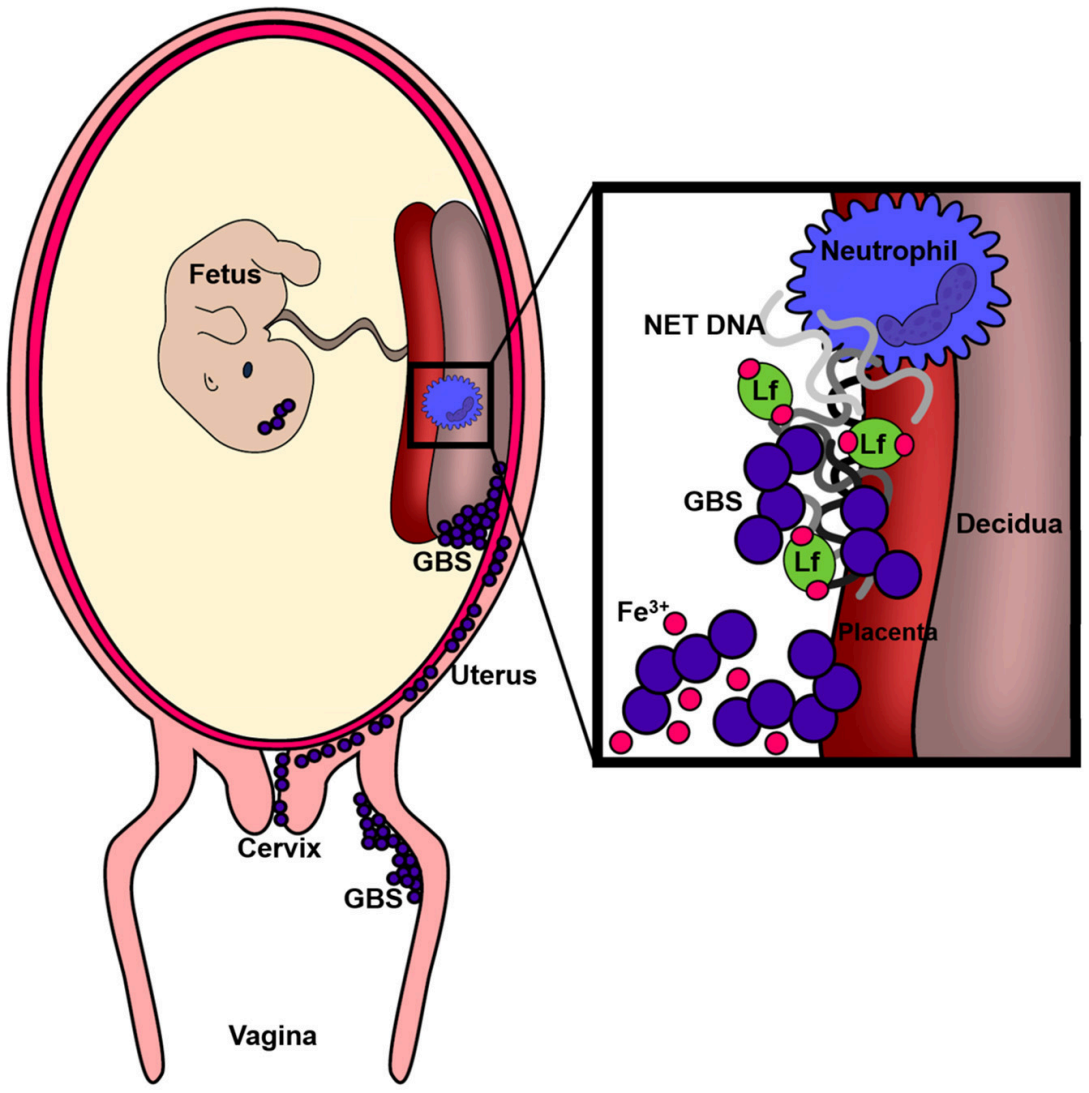
**Conceptual diagram of the murine model of GBS infection during pregnancy**. Results derived from *in vivo* and *ex vivo* experiments reveal GBS has the capacity to cause ascending vaginal infection during pregnancy, induction of neutrophil recruitment to invaded tissues, and that GBS-neutrophil interactions promote extracellular trap formation and deposition of antimicrobial molecules (such as lactoferrin) that promote nutritional immunity.

In conclusion, we report that a pregnant mouse model of ascending GBS vaginal infection results in bacterial colonization of the reproductive tract and invasion of the placenta, decidua and fetus (See Figure 9). As a consequence of GBS invasion, neutrophils are recruited to the placental and decidual compartments; likely as a host strategy to contain the infection. In response to GBS infection, lactoferrin is elevated within the placental and decidual tissue. Furthermore, *ex vivo* results indicate neutrophils elaborate extracellular traps decorated with lactoferrin upon encountering GBS. Lactoferrin can subsequently exert an antimicrobial activity *in vitro* against GBS via chelation of nutrient iron, which is required for GBS growth and viability. Thus, a better understanding of lactoferrin deposition by neutrophils in NETs and the interplay of this important host molecule with GBS could lead to novel chemotherapeutic strategies as the utility of antibiotics wanes.

## Author contributions

VK, RD, LR, LK, KB, JR, KH, and JG performed the experiments. SM, JG, and DA conceptualized experiments and analyzed data. VK and JG wrote the manuscript. All authors read and edited the manuscript.

### Conflict of interest statement

The authors declare that the research was conducted in the absence of any commercial or financial relationships that could be construed as a potential conflict of interest.

## Supplementary material

Supplemental Figure 1 **Immunohistochemical and immunopathological analyses of uninfected or GBS-infected vaginal muscularis and mucosal tissue**. Immunopathological examination by hematoxylin and eosin staining (H&E) reveal neutrophils at the surface of the mucosa (black arrows, inset panel of GBS-infected tissue micrograph), which corresponds to an area within the mucosa that is colonized by GBS as determined by immunohistochemical staining with a polyclonal rabbit antibody to GBS (white arrows, inset panel of GBS-infected tissue micrograph), a result that was not observed in uninfected tissue (Uninfected).

Supplemental Figure 2 **Immunohistochemical and immunopathological analyses of uninfected or GBS-infected uterine tissue (myometrium, endometrium, and lumen)**. Immunopathological examination by hematoxylin and eosin staining (H&E) reveal preserved tissue architecture in both uninfected and GBS-infected animals. However, immunohistochemical staining indicates GBS colonizes the lumen and the surface of the endometrium as determined by staining with a polyclonal rabbit antibody to GBS (white arrows, inset panel of GBS-infected tissue micrograph), a result that was not observed in uninfected tissue (Uninfected).

Supplemental Figure 3 **Gating strategy for flow cytometry analyses of neutrophils isolated from mouse reproductive tissues**. Neutrophils were stained with antibodies specific to 1A8 GR1 clone (Ly6g), CD45, and Neu7/4, and analyzed by flow cytometry using a gating strategy to quantify cells defined as Neu7/4^high^, GR1^high^ within the viable CD45+ cells (27.9% of Viable CD45+ cells). In comparison, similar results were obtained when neutrophils were stained with antibodies specific to 1A8 GR1 clone (Ly6g), CD45, and CD11b, and analyzed by flow cytometry using a gating strategy to quantify cells defined as CD11b^high^, GR1^high^ within the viable CD45+ cells (29.8% of Viable CD45+ cells).

Supplemental Figure 4 **Iron chelation inhibits GBS growth**. Spectrophotometric analyses reveal increasing concentrations of the synthetic iron chelator 2, 2′ dipyridyl (Dipyridyl) repress GBS cell density in medium alone (Medium Alone) after 24 h of culture. Supplementation with excess exogenous nutrient iron (250 μM ferric chloride, Medium + Iron) restores bacterial growth in the presence of the synthetic chelator (*P* < 0.05, Student's *t*-test comparing medium alone to medium + iron), indicating iron is a critical micronutrient for GBS growth and proliferation.
