# Supplementary figures and images for: Group B Streptococcus Induces Neutrophil Recruitment to Gestational Tissues and Elaboration of Extracellular Traps and Nutritional Immunity
Source: Front Cell Infect Microbiol. 2017 Feb 3;7:19. doi: 10.3389/fcimb.2017.00019 (PMC5289994; doi:10.3389/fcimb.2017.00019)

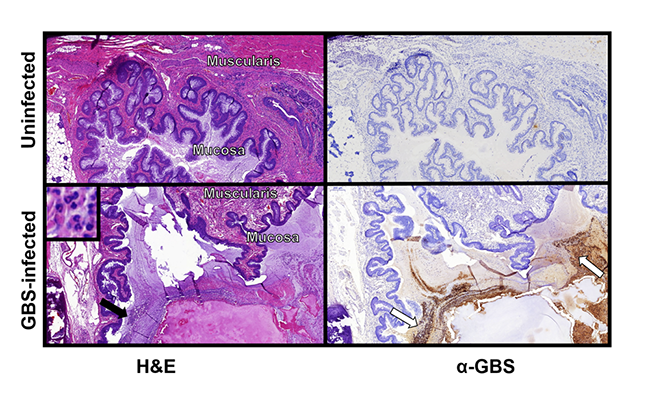

Supplement: Supplemental Figure 1 — Immunohistochemical and immunopathological analyses of uninfected or GBS-infected vaginal muscularis and mucosal tissue. Immunopathological examination by hematoxylin and eosin staining (H&E) reveal neutrophils at the surface of the mucosa (black arrows, inset panel of GBS-infected tissue micrograph), which corresponds to an area within the mucosa that is colonized by GBS as determined by immunohistochemical staining with a polyclonal rabbit antibody to GBS (white arrows, inset panel of GBS-infected tissue micrograph), a result that was not observed in uninfected tissue (Uninfected). [file Image1.tif]

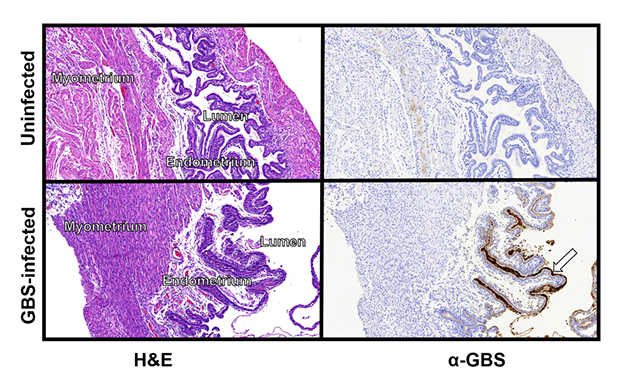

Supplement: Supplemental Figure 2 — Immunohistochemical and immunopathological analyses of uninfected or GBS-infected uterine tissue (myometrium, endometrium, and lumen). Immunopathological examination by hematoxylin and eosin staining (H&E) reveal preserved tissue architecture in both uninfected and GBS-infected animals. However, immunohistochemical staining indicates GBS colonizes the lumen and the surface of the endometrium as determined by staining with a polyclonal rabbit antibody to GBS (white arrows, inset panel of GBS-infected tissue micrograph), a result that was not observed in uninfected tissue (Uninfected). [file Image2.tif]

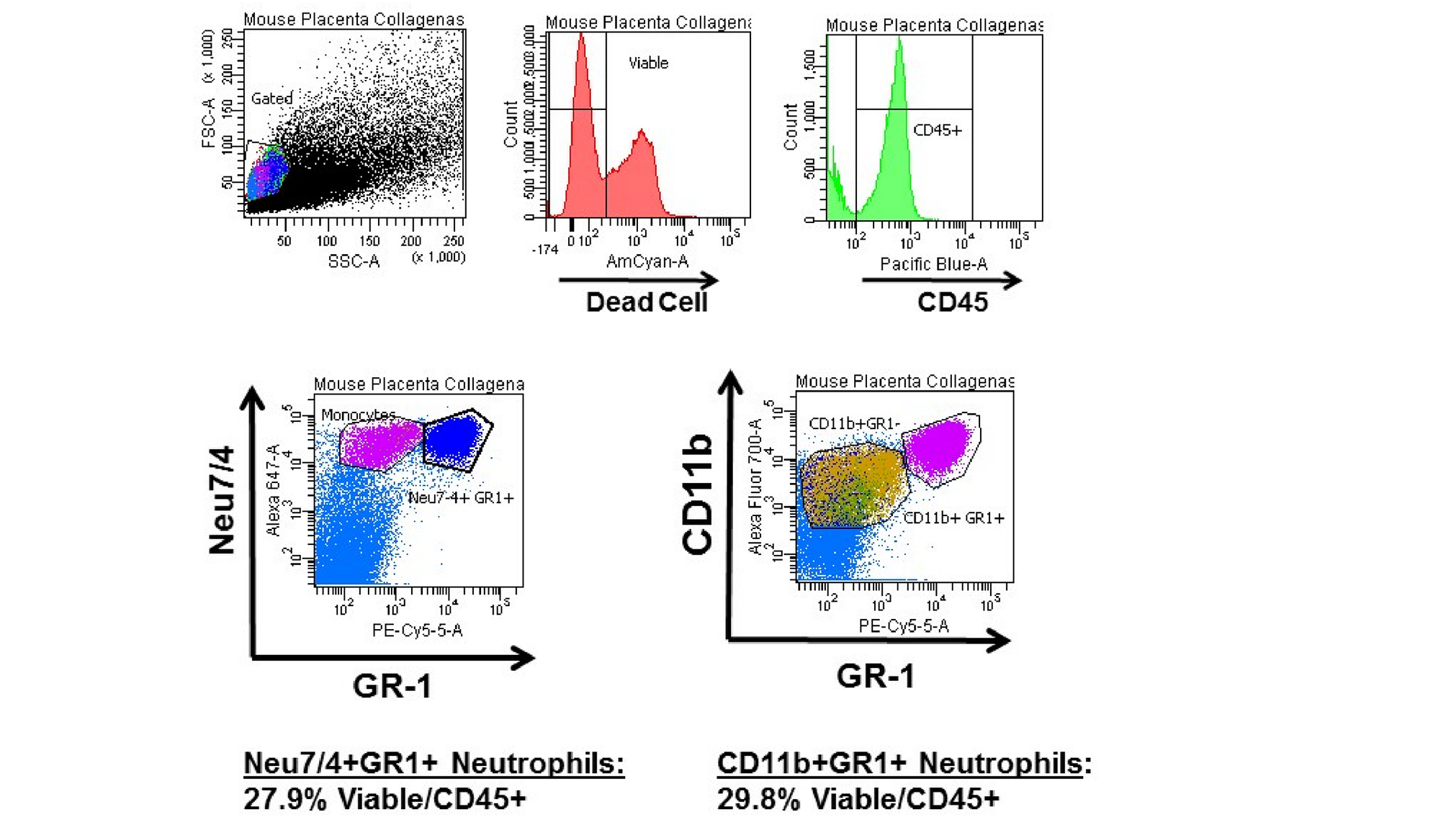

Supplement: Supplemental Figure 3 — Gating strategy for flow cytometry analyses of neutrophils isolated from mouse reproductive tissues. Neutrophils were stained with antibodies specific to 1A8 GR1 clone (Ly6g), CD45, and Neu7/4, and analyzed by flow cytometry using a gating strategy to quantify cells defined as Neu7/4high, GR1high within the viable CD45+ cells (27.9% of Viable CD45+ cells). In comparison, similar results were obtained when neutrophils were stained with antibodies specific to 1A8 GR1 clone (Ly6g), CD45, and CD11b, and analyzed by flow cytometry using a gating strategy to quantify cells defined as CD11bhigh, GR1high within the viable CD45+ cells (29.8% of Viable CD45+ cells). [file Image3.TIF]

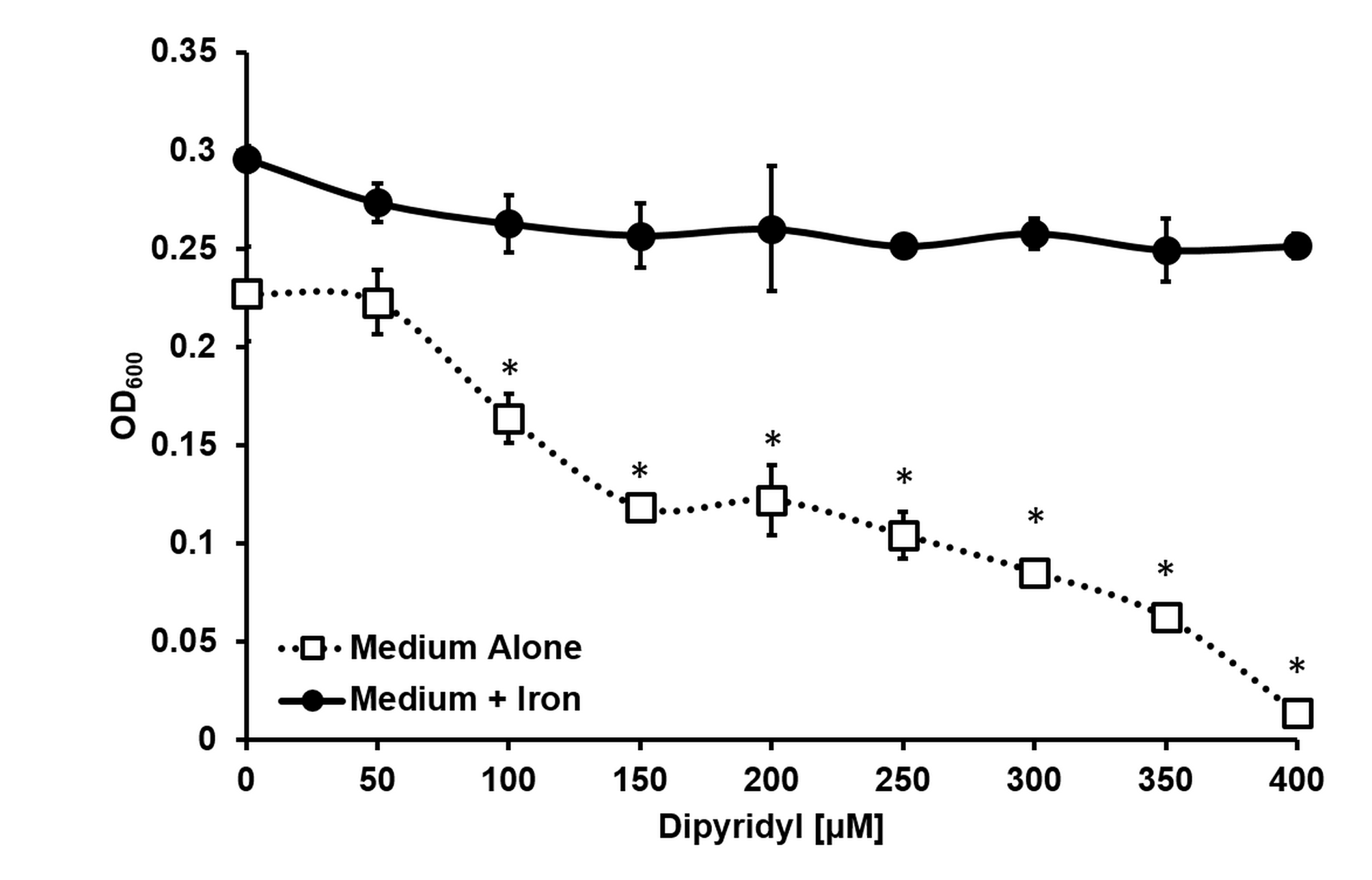

Supplement: Supplemental Figure 4 — Iron chelation inhibits GBS growth. Spectrophotometric analyses reveal increasing concentrations of the synthetic iron chelator 2, 2′ dipyridyl (Dipyridyl) repress GBS cell density in medium alone (Medium Alone) after 24 h of culture. Supplementation with excess exogenous nutrient iron (250 μM ferric chloride, Medium + Iron) restores bacterial growth in the presence of the synthetic chelator (P < 0.05, Student's t-test comparing medium alone to medium + iron), indicating iron is a critical micronutrient for GBS growth and proliferation. [file Image4.TIF]
